# Comparison of quality and microstructure of chokeberry powders prepared by different drying methods, including innovative fluidised bed jet milling and drying

**DOI:** 10.1007/s10068-019-00556-1

**Published:** 2019-04-06

**Authors:** Anna Sadowska, Franciszek Świderski, Rita Rakowska, Ewelina Hallmann

**Affiliations:** Department of Functional and Organic Food and Commodities, Faculty of Human Nutrition and Consumer Sciences, Warsaw University of Life Sciences, Nowoursynowska Str. 159c, 02-776 Warsaw, Poland

**Keywords:** Drying method, Chokeberry, Powders

## Abstract

The study assessed the functional properties, microstructural features and sensory characteristics of chokeberry powders obtained by the new fluidised bed jet milling and drying method as compared to other drying methods such as freeze-drying, vacuum drying and convection drying. The new method was based on simultaneously drying and milling plant material which had been subjected to partial air drying at 70 °C prior to this treatment. The technique resulted in the production of good quality fruit powders with high vitamin C and polyphenolic content, as well as high antioxidant activity. Favourable physiochemical characteristics and sensory qualities were also observed together with low water activity. The new fluidized bed jet milling and drying therefore compared very favourably with freeze-drying, vacuum drying and convection drying when considering the content of bioactive substances, physiochemical features and the sensory quality of the prepared powders.

## Introduction

The black chokeberry (*Aronia melanocarpa* (Mixch.) Elliott) is a native North American shrub belonging to the *Rosaceae* family which was introduced into Europe. Chokeberries are a good source of polyphenols, which exhibit high antioxidant activity. The main group of polyphenols found in this plant are anthocyanins, which are responsible for the astringent taste and intense colouration (Caruso et al., 2016; Kulling and Rawel, 2008). Many recent studies have demonstrated multidirectional positive effects of these compounds on health. Anthocyanins positively influence the circulatory system and the functioning of the heart. They stimulate the secretion of insulin and improve the functioning of the retina (Bermúdez-Soto et al., 2007; Lala et al., 2006; Li et al., 2017). However, chokeberries are not usually eaten raw due to their astringent taste and thus need to be suitably processed to be acceptable for general consumption as either partially finished or finished products. With such processed products, preparing the dried form of the fruit using convection or sublimation and then milling to suitable degrees of powder is essential. This then enables their inclusion in health-promoting products such as fruit teas, ready-made fruit juices, fruit yoghurts, desserts and dietary supplements. Vital bioactive ingredients may however be lost whenever fruit is dried, particularly polyphenols and vitamin C, and therefore it is very important that appropriate drying and milling methods are used to obtain powdered forms. A cost effective method has been convective drying (CD) which is still widely used in order to obtain dried forms of a given product. However, this method requires long drying times and high temperatures that degrade vital nutrients along with changing the colour (Soysal et al., 2009). In order to obtain both high quality and nutritional value in dehydrated fruit and vegetables, a freeze drying (FD) technique has been employed. Nevertheless, it is an expensive and very slow process because of the low drying rates required resulting in relatively low yields and high energy costs due to the need to use frozen and vacuum systems (Nireesha et al., 2013). As the FD method consumes a lot of energy, this seriously limits its wider application. Much focus has been given recently to adopting new methods of drying and milling hard materials for producing powdered fruit and vegetables. There are known methods of using fluid-energy for drying solid products including the fluidised-bed driver and its modification, the pulsed fluidised-bed, in which gas pulses cause vibration of the particle bed. For micronising products the s-Jet system can be used, and is primarily recommended for nano application minerals, pharmaceuticals and cosmetics (Gawrzynski et al., 1999). The related fluidised-bed jet milling and drying (FBJD) technology and the s-Jet system used in this study employ fluid-energy impact milling for both milling and drying of the raw material. Moisture from the micronised sample is rapidly evaporated through a strong air flow achieved either by a high energy air stream (the FBJD method) or by preheated steam (the s-Jet method) upon being introduced into the milling compartment. High-energy particles arising from their mutual collisions in the carrier gas stream (air, inert gas or steam) are filtered to a desired particle size. Evaporation from the particulate raw material sample can occur over a wide temperature range, even at those below 40 °C. Pre-drying of the raw material is thus recommended for the sake of efficiency. Nutritional values of manufactured foodstuffs can be retained to a large degree through combining the milling and drying stages in a strong and high-energy stream of air (or inert gas), where drying can be achieved at lower temperatures. Previous and preliminary studies by our group using this method on chokeberry and kale powder samples demonstrated great promise because powders so obtained had both high sensory qualities and amounts of bioactive components (Sadowska et al., 2017a). This study is a continuation of the above described and is devoted to assessing the following parameters of quality: amounts of bioactive substances, antioxidant potential, physiochemical and sensory characteristics together with those micro-structural features of chokeberry powders obtained by the new FBJD with pre-drying as compared to other drying methods such as freeze-drying (FD), vacuum drying (VD) and convection drying (CD).

## Materials and methods

### Materials

The tested materials consisted of chokeberry powders obtained by the following methods: FBJD, FD, VD and CD. Milled powders were obtained after the fruit had been dried by FD, VD and CD. Evimax Vega (Radom, Poland) provided the FBJD samples from their experimental industrial manufacturing plant which used the new method of simultaneous drying and milling (Sadowska et al., 2017a). The pre-drying temperature of 70 °C was chosen for reducing the water content by 25% at a flow rate of 50 m/s. Due to mutual particle collision in the high-energy air stream the test material particles were crushed. Those dried powders with a water content < 5% were carried by the air stream to a vibration classifier so that powders of predetermined fragment sizes could be obtained. The product yield was 50 kg/h.

Samples for FD were first frozen at − 30 °C for 48 h, and freeze drying was performed by the Donserv Liofilizator Alpha model 1–4 LSC (Donserv, Warsaw, Poland) at − 50 °C, 10 Pa pressure and shelf temperature of 21 °C. Vacuum drying was accomplished in an SPT-200 vacuum chamber dryer (Conbest Company, Cracow, Poland) linked to a Mensor mass transfer system. Drying was carried out until stable mass readings were obtained within 30 min at a temperature of 65 °C and 4 kPa pressure in the vacuum dryer compartment. Convection drying was performed using a laboratory convection dryer (Wamed Company, Warsaw, Poland) at 70 °C for 48 h. After the FD, CD and VD drying processes, the dried sample test material was milled by a grinding knife-type device. The powders obtained were packed in moisture-proof packaging and then used for our study as the subject material. The particle size of powders dried by different methods was controlled by screening and was less than 400 μm.

### Preparation of extracts for antioxidant properties and total polyphenol content analysis

A 250 mg quantity of the tested dried sample was weighed (with 0.001 g accuracy) into sterile, plastic, FALCONE-type test tube with a screw top (50 mL capacity) and 25 mL of distilled water was added. It was shaken in a vortex mixer LP Vortex (Labo Plus, Warsaw, Poland) for 60 s in order to mix it thoroughly and then incubated in an incubator with a vortex (KS 4000 i Control, IKA, Staufen, Germany) for 60 min at 30 °C. After the incubation process, the sample was shaken again in a vortex for 60 s to receive a thorough mixing, and then spun in a centrifuge with a cooling system (MPW-380 R, MPW Med. Instruments, Warsaw, Poland) (5 °C, 12076×*g*, 20 min).

### Antioxidant activity

Antioxidant activity in water extracts of the test material was determined by using ABTS+· (2,2′-azino-bis-3-ethylbenzothiazoline-6-sulphonic acid) radical cation assay according to the modified method of Re et al. (1999). A certain quantity of the tested extracts’ solution, which was determined earlier by a designated dilution scheme, was drawn into 10 mL glass test tubes and 3.0 mL of radical cations’ ABTS+· in PBS solution was added. The absorbance measurement was made after exactly 6 min of incubation at room temperature. The absorbance was measured after exactly 734 nm, with the use of a spectrophotometer (UV/Vis UV-6100A, Metash Instruments Co., Ltd, Shanghai, China). The results were represented as as mmol of Trolox per 100 g of dm.

### Total polyphenol and anthocyanins contents

The total content of polyphenols in water extracts of the test material was determined by using the Folina–Ciocalteu (F–C) reagent, according to the modified method of Singleton and Rossi (1965). A certain quantity of the tested extracts solution, which was determined earlier by a designated dilution scheme, was drawn into 50 mL flasks, then, 2.5 mL of the F–C reagent and 5.0 mL of 20% sodium carbonate were added, and finally made up to volume with distilled water. The samples were incubated for 60 min at room temperature with no light access. The absorbance was measured at 720 nm, with the use of a spectrophotometer (UV/Vis UV-6100A, Metash Instruments Co., Ltd). The results were represented as mg GAE (Gallic Acid Equivalent) in 100 g of tested powder (dry weight).

The anthocyanin content (cyanidin-3,5-digalactoside and cyanidin-3,5-diarabinoside) was determined using the HPLC method described by Hallmann (2012). A sample of 100 mg powder was weighed into a plastic test tube, then 1 mL of methanol with 1% ascorbic acid was added. The solution was mixed thoroughly by vortexing and incubated in an ultrasonic bath (15 min at 30 °C). The samples were then spun at 5000 rpm. From the test tube 1 mL of extract was collected and respun at 12,000 rpm. An aliquot of 500 μL of extract was taken for analysis by HPLC. Anthocyanins were determined by using the HPLC with a Synergi Fusion-RP 80i column (250 × 4.60 mm) (Phenomenex, Torrance, California, USA), and elution was by using gradient flow with two mobile phases: acetonitrile/deionised water (55% and 10%), at pH 3.00. The analysis time was 36 min, the flow rate was 1 mL/min, and the wavelength range for detection was 250–370 nm. Anthocyanins were identified based on Sigma Aldrich (Saint Louis, Missouri, USA) external standards (cyanidin-3,5-digalactoside (99.0%) and cyanidin-3,5-diarabinoside (99.0%)). The Shimadzu HPLC (USA Manufacturing Inc., Oregon, USA) was used, consisting of two LC-20AD pumps, a CMB20A system controller, SIL-20AC autosampler, UV/VIS SPD-20AV detector, and CTD-20AC controller. The results are expressed in mg of total content of anthocyanins/100 g d.m.

### Vitamin C

Vitamin C was measured as the sum of ascorbic acid and dehydroascorbic acid by HPLC with UV detection (Waters, Milford, Massachusetts, USA) at a 245 nm wavelength and mobile phase flow rate of 0.8 mL/min. Total vitamin C sample content was determined following extraction for ascorbic acid and dehydroascorbic acid and then reduction using the dithiothreitol reagent. HPLC was then performed with a UV 2487 detector and separation by an RP Symmetry C18.5 µm. 4.6 × 150 mm column at a temperature of 25 °C, where the injection volume varied between 10 and 30 µL.

### Water holding capacity (WHC)

The WHC was determined according to the procedure of Sudha et al. (2007). In a centrifuge tube, 1 g of powder was mixed with 50 mL of distilled water, centrifuged at 10,000 rpm (MPW-380 R, MPW Med. Instruments) for 15 min, and the supernatant was decanted. The powder with absorbed water was reweighed, and WHC was expressed as g water/g d.m.

### Water solubility index (WSI)

The WSI was measured using the method of Anderson et al. (1969). A mass of 2.5 g of powder and 30 mL distilled water were vigorously mixed in a 100 mL centrifuge tube, incubated in a 37 °C waterbath for 30 min and then centrifuged for 20 min at 10,000 rpm (MPW-380 R, MPW Med. Instruments). The supernatant was carefully collected in a pre-weighed beaker and oven-dried at a temperature of 103 ± 2 °C. The WSI was calculated as the percentage of dried supernatant (g) relative to the 2.5 g powder.

### pH

The pH was measured using a laboratory pH-meter (CP-511, Elmetron, Zabrze, Poland).

### Colour

Powder colour was determined by the Minolta Chroma Meter instrument (Model CR-400, Konica Minolta Inc., Tokyo, Japan) with the L*a*b* measuring system (illuminant D65.2° standard observer, measurement area 8 mm). Values for L* (lightness), a* (− greenness, + redness) and b* (− blueness, + yellowness) were determined.

### Dry matter

Dry matter was measured by a gravimetric method according to AOAC (2005) methodology.

### Water activity (Aw)

The Aw was measured using a manual AquaLab Water Activity Meter (Decagon Devices. Inc., Pullman, Washington, USA).

### Microstructure

The microstructure of the chokeberry powders was investigated using a scanning electron microscope Quanta 200 XL series (Thermo Fisher Scientific, Hillsboro, Oregon, USA).

### Sensory analysis

Sensory characteristics of the drinks prepared from the powder samples were determined by Quantitative Descriptive Analysis (QDA) in accordance with the regulatory procedure described in ISO 13299:2003. Nineteen (19) quality parameters were selected for analytical profiling of the powder drinks which had been prepared at a 5% concentration of each powder in 5% water and sugar solution. The quality parameters were: smell (fruity, sweet, sour, sharp, other), taste (fruity, sweet, sour, bitter, tart, other), colour, homogeneous appearance, density, smoothness, chalkiness, powder perceptibility, powder residue and overall quality. These sensory characteristics were evaluated by a 9-person team of qualified assessors who were experts according to PN-EN ISO 8586:2014. The sensory evaluation was performed in two independent replicates. Ratings were performed at the Laboratory of Sensory Analysis which meets all criteria specified in the BS EN ISO 8589:2010. standard. The computerised support system for sensory analysis (Analsens NT, Caret Systemy Cyfrowe i Oprogramowanie Sp. z o.o., Gdańsk, Poland) was used to plan sessions with the ratings scaling method as well as for generating random numbers for sample coding and keeping records of individual results and their pre-treatments.

### Statistical analysis

Statistica 10.0 (Statsoft, Stat Soft Power Solutions, Inc., Tulsa, Oklahoma, USA) software was used for all statistical processing. A ‘One-Way-Analysis of Variance’ (ANOVA) was carried out to test for significant differences in the quality characteristics between the compared powders, using the Duncan significance test for post hoc testing between groups at *α* = 0.05. Principal component analysis (PCA) was used to assess the similarities and differences amongst the sensory characteristic of the powders that were evaluated according to a covariance matrix.

## Results and discussion

### Bioactive component content and antioxidant potential

Vitamin C content in chokeberry powders prepared by FBJD and FD, Table 1, was similar to those found in fresh fruit (81.87 ± 6.87 mg/100 g d.m.). However significant differences were found between powders prepared by VD and CD, where vitamin C losses were 24% and 59%, respectively. In a previous study by the Sadowska et al. (2017a), vitamin C contents were significantly reduced by approximately 29% in chokeberry powders obtained through FBJD and FD methods as compared to fresh fruit samples. Such different outcomes for FBJD-prepared samples in the first and second studies may have arisen because of the lower pre-drying temperature of 50 °C used in the first study compared to the 70 °C used in the present study. A study by Sadowska et al. (2017b) showed significantly lower levels of vitamin C in CD chokeberries dried for 48 h at 50 °C with forced air circulation as compared to FD. Vitamin C levels in the raw fruit and in the dried and lyophilised samples were in fact several times lower than in the present study. Such differences could be put down to the different types of tested chokeberries. A study by Marques and Freire (2006) found that FD resulted only in small losses of vitamin C for tropical fruit, whilst Lin et al. (1998) demonstrated that vitamin C levels in carrots did not differ between FD and air-drying methods, with both reducing the vitamin C content by 62%. Bober and Oszmianski (2004) reported losses of vitamin C for chokeberry pomace at 60% after FD and 80% after CD. The highest levels of polyphenolic content and sum of two main anthocyanins were achieved when using the FBJD method, Tables 1 and 2. A previous study by our group (Sadowska et al., 2017a) showed higher levels of polyphenols and anthocyanins in FD chokeberry powders compared to the FBJD method. This may have been due to the lower pre-drying temperature (50 °C) used for CD compared to FBJD. Another study by Sadowska et al. (2017b) found two-fold losses in chokeberry powder anthocyanins when dried by CD as compared to FD. Our study has presented the two principal anthocyanin compounds, cyanidin-3,5-diarabinoside and cyanidin-3,5-digalactoside which accounted for approximately 50% of the total polyphenol content as compared to the results of the polyphenols content obtained using the spectrophotometric method. The different drying methods gave varying levels of these anthocyanins which is linked to their high sensitivity to temperature and the presence of oxygen. The lowest amounts of cyanidin-3,5-diarabinoside and cyanidin-3,5-digalactoside were found after CD (around 421 mg/100 g d.m.), whereas the highest amounts were found after FBJD (around 1087 mg/100 g d.m.) and FD (around 904 mg/g d.m.). In the study by Samoticha et al. (2016), anthocyanin losses ranged from 43 to 80% according to the drying method; the highest (80%) was for CD drying at 60 °C, and the lowest for the FD method 43%), with VD between the two (53%). Wojdylo et al. (2009) found 73% losses of these compounds during the CD drying of strawberries. Losses of anthocyanins in Saskoton berries amounting to 83–88% after CD (at 73 °C) and 23–32% after FD were found by Kwok et al. (2004). The present study demonstrated similarly high levels (58.4–58.9 mmol Trolox/100 g d.m.) of antioxidants in chokeberry powders obtained by both the FBJD and FD methods, however significantly lower levels of antioxidant activity were found after VD and CD, Table 1. Previous studies by Sadowska et al. (2017a) showed lower antioxidant activity in chokeberry powder after FBJD than FD; likewise the same was found for polyphenols which, as aforementioned, might be due to the lower pre-drying temperatures adopted for the latter. A study by Horszwald et al. (2013) showed significantly higher ability for neutralising ABTS+· radicals when chokeberry powders were obtained by the FD method compared to dried VD powders at different temperatures. The study by Samoticha et al. (2016) reported higher antioxidant activities and polyphenol content for FD and VD in dried chokeberries whilst after CD, values were significantly lower compared to the presented work; this might be due to the different drying parameters used in these studies. The decrease in the antioxidant activity of chokeberry powders was a result of the loss both of anthocyanins and vitamin C content. However, considering the high content of anthocyanins in chokeberry and the relatively low content of vitamin C, it could be said that anthocyanins should exert a much greater influence on the antioxidant properties of chokeberry than does vitamin C. In numerous studies, including Aliakbarlu et al. (2014) and Surma et al. (2013), a high positive correlation between the content of polyphenols and the antioxidant activity of the tested products was obtained. In the study by Gawlik-Dziki (2004), it was proved that in addition to polyphenols, antioxidant properties may be dependent on the presence of compounds such as ascorbic acid, carotenoids or α-tocopherol. Taking into account the results obtained, it can be concluded that the FBJD and FD powders were characterized by the highest content of bioactive substances and antioxidant properties as compared to the CD and VD powders. This may be related to the longer drying time in the case of CD and VD methods and the mechanical grinding of the dried material, during which it is exposed to atmospheric oxygen.

**Table 1:** Vitamin C and polyphenol levels and antioxidant properties in chokeberry powders obtained by FBJD, FD, VD and CD methods (average values ± standard deviation)

Drying method	Vitamin C content mg/100 g d.m.	Polyphenol contents mg GAE/100 g d.m.	Antioxidant activity mmol Trolox/100 g d.m.
FBJD	80.57^c^ ± 7.77	2484.60^b^ ± 83.32	58.91^c^ ± 1.32
FD	79.84^c^ ± 5.96	2255.86^a^ ± 75.39	58.36^c^ ± 1.86
VD	62.14^b^ ± 3.31	2148.82^a^ ± 145.99	55.63^b^ ± 0.59
CD	32.99^a^ ± 6.23	2147.17^a^ ± 89.61	48.95^a^ ± 0.83

a–c—superscripts indicate significant difference in columns between means (*α *= 0.05)

*FBJD* fluidised bed jet drying, *FD* freeze-drying, *VD* vacuum drying, *CD* convection drying

**Table 2:** Anthocyanin (mg/100 g d.m.) levels in chokeberry powders obtained by FBJD, FD, VD and CD methods (average values ± standard deviation)

Drying method	Cyanidin-3,5-digalactoside	Cyanidin-3,5-diarabinoside	Sum
FBJD	1035.51^c^ ± 40.25	51.48^b^ ± 5.02	1086.99^c^ ± 51.11
FD	766.59^b^ ± 17.03	137.63^d^ ± 24.98	904.23^b^ ± 41.45
VD	769.11^b^ ± 55.37	76.47^c^ ± 3.16	845.58^b^ ± 53.21
CD	397.71^a^ ± 4.67	23.87^a^ ± 0.57	421.58^a^ ± 2.60

a–d—superscripts indicate significant difference in columns between means (*α *= 0.05)

*FBJD* fluidised bed jet drying, *FD* freeze-drying, *VD* vacuum drying, *CD* convection drying

### Physiochemical properties of the prepared chokeberry powders

**Fig. 1:**
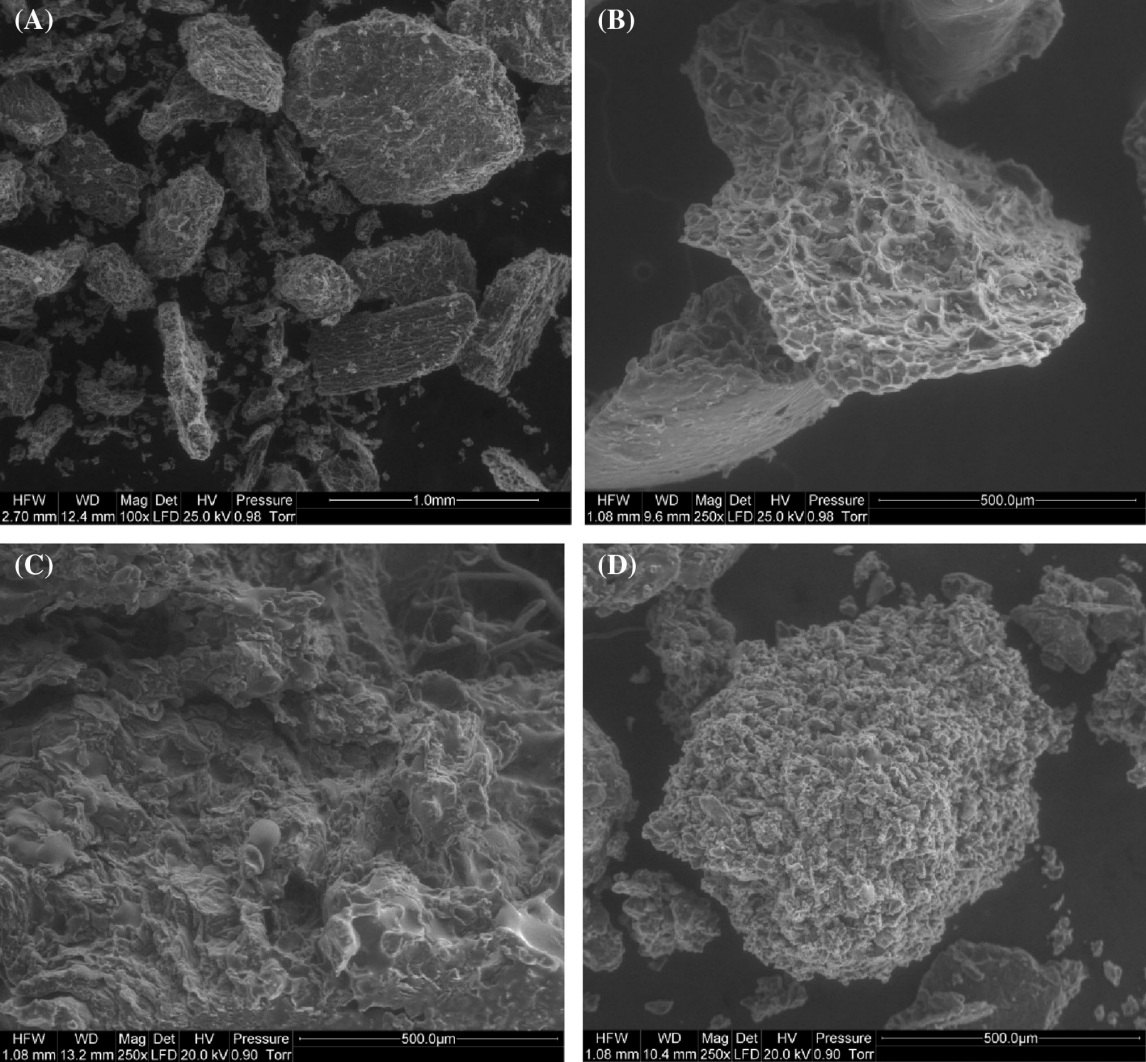
Microstructure of chokeberry powders by scanning electron microscopy (A) fluidised bed jet drying (FBJD), (B) freeze-drying (FD), (C) vacuum drying (VD), (D) convection drying (CD)

All tested powders had very low water activity and dry mass, which should ensure microbiological stability during long storage periods in moisture barrier packaging, Table 3. A safe water activity value for raw products is below 0.600, which may reduce microbial growth, including that of osmophilic yeast (Samoticha et al., 2016). The tested powders had comparable WHC properties (2.73–2.95%) and varying WSI properties: the values ranged from 48.43% for CD to 61.65% for VD powders, while WSI properties obtained for FD and FBJD powders varied from 57.00 to 57.85%, Table 3. The WHC and WSI results might have resulted from differences in the microstructure of the powders that were created by the comminution of the dried fruit using various methods, as shown in Fig. 1. The similarities in WHC values could be explained by the fact that the process of intense milling of chokeberry fruits that had been dried with different methods created particles of similar size and it destroyed the majority of the fruits’ primary structure. At the same time, the powders had a relatively large surface area, which allowed them to absorb water particles to a comparable degree, regardless of the method. In the case of whole lyophilised fruits that had not been subjected to comminution, the spongy texture could absorb up to 2–4 times more water than the fruits that had been processed by convection drying—their microstructure has been considerably damaged (Ratti, 2001). Based on microscopic structural examination of the dried material it was observed that cellular structure becomes disrupted and contracted during CD, thereby leading to the product being poorly restored. The low WSI values obtained for CD powders could have resulted from the compressed, hard microstructure of the powders, since separation and migration of minuscule particle aggregates was made relatively difficult. VD method produced powders with a looser microstructure (they were not as compressed), Fig. 1C—it was associated with a lower drying temperature combined with a drying period that was significantly shorter than the one used in CD. Hence the WSI values for these powders were higher. The comparable WSI values for FD and FJDB powders could have been the results of a less compressed powder structure and a partially retained spongy texture, Fig. 1A, B, which made it easier for particle aggregates to separate in comparison to CD powders, and more difficult in comparison to VD powders. The authors’ previous findings confirm the results for chokeberry fruit powders, obtained by FD and FJDB methodology, while higher values were obtained for FD method (Sadowska et al., 2017a).

**Table 3:** Water activity, dry matter, WHC, WSI and pH in chokeberry powders obtained by FBJD, FD, VD and CD methods (average values ± standard deviation)

Drying methods	Water activity	Dry matter (%)	WHC (g H_2_O/g d.m.)	WSI (%)	pH
FBJM	0.1359^b^ ± 0.14	97.53^a^ ± 0.43	2.95^a^ ± 0.48	57.85^b^ ± 1.66	4.27^c^ ± 0.03
FD	0.0959^a^ ± 0.27	99.18^c^ ± 0.14	2.94^a^ ± 0.25	57.00^b^ ± 1.83	3.89^a^ ± 0.01
VD	0.1301^b^ ± 0.43	98.21^b^ ± 0.18	2.73^a^ ± 0.85	61.65^c^ ± 0.08	3.96^b^ ± 0.03
CD	0.1625^c^ ± 0.68	97.18^a^ ± 0.27	2.95^a^ ± 0.48	48.43^a^ ± 0.48	4.25^c^ ± 0.02

a–c—superscripts indicate significant difference in columns between means (*α *= 0.05)

*FBJD* fluidised bed jet drying, *FD* freeze-drying, *VD* vacuum drying, *CD* convection drying

Significant differences in all colour parameters were noted for all powders according to drying methods, Table 4. The FBJD chokeberry powder had higher values of the L*, b* and a* colour parameters, indicating that this powder was lighter and had more intense red and yellow colours than the others. Fruit colouring is both a reflection of the type and amount of colour compounds therein and is also characteristic of any given variety (Ochmian et al., 2012). The colour of chokeberries is chiefly dependent on anthocyanin content (Konczak and Zhang, 2004). The FBJD powder had higher amounts of anthocyanins and, at the same time, demonstrated a more intense red colouration (i.e. a higher a* colour value). Differences in the colours of the powders arose mostly from the various techniques used in powder milling. The FBJD powder was crushed throughout drying in a sealed chamber, whilst the FD, VD and CD powders were milled by a grinding mill exposed to air. This could result in fewer red and yellow powder colours in the samples exposed to air than in the FBJD powder.

**Table 4:** Colour parameters in chokeberry powders obtained by FBJD, FD, VD and CD methods (average values ± standard deviation)

Drying method	Colour parameters		
	L*(D65)	a*(D65)	b*(D65)
FBJD	18.10^b^ ± 0.24	9.20^d^ ± 0.18	4.57^c^ ± 0.11
FD	15.09^a^ ± 0.64	8.85^c^ ± 0.05	2.64^b^ ± 0.04
VD	15.25^a^ ± 2.16	5.96^b^ ± 0.17	1.64^a^ ± 0.45
CD	15.05^a^ ± 0.02	4.75^a^ ± 0.08	1.76^a^ ± 0.18

a–d—superscripts indicate significant difference in columns between means (*α *= 0.05)

*FBJD* fluidised bed jet drying, *FD* freeze-drying, *VD* vacuum drying, *CD* convection drying

In conclusion, it can be stated that the powders obtained using the new methodology were characterised by physicochemical properties similar to powders obtained by FD and VD methods, and better than those obtained by CD.

### Sensory evaluation

**Fig. 2:**
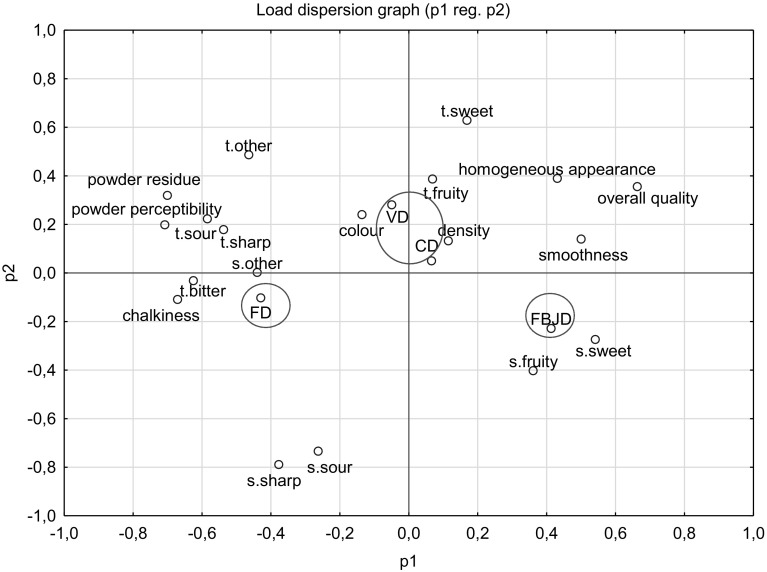
Similarities and differences found in the sensory qualities of the tested chokeberry drinks prepared at a 5% concentration of powders in 5% sugared water. FBJD—fluidised bed jet drying; FD—freeze-drying; VD—vacuum drying; CD—convection drying

Figure 2 depicts the relationship between the sensory characteristics of the chokeberry drinks prepared at a 5% concentration of tested powders in 5% sugared water and the main components. This projection distributes the results on the plane formed by the two selected factors responsible for sample variability; i.e. the projection constitutes a correlation image of the sensory data. The graphical positioning of the chokeberry samples within the coordinate system constructed from the two main components demonstrates that the evaluated sensory parameters were diverse. The tested chokeberry drinks in the projection were divided into three groups: FD, FBJD and VD/CD. They were positioned quite distinctly from each other meaning that the evaluated sensory parameters differed quite markedly. The chokeberry products obtained by VC and CD had very similar sensory parameters, including colour, fruity taste, density and sweet taste, thus they are positioned close to each other. The FBJM chokeberry drinks were more intensely fruity and sweet as well as being smoother, having a more homogeneous appearance and higher overall quality descriptions compared to the FD chokeberry drinks. The FD drinks were characterised by bitter, sour and sharp tastes, were chalkier when compared to the VD, CD and FBJD drinks, and had more intense powder residue and perception. Such variations in the consistencies of the drinks (e.g. smoothness and homogeneous appearance) can be accounted for by the powders’ microstructure, Fig. 1.
